# Historical Underpinnings of Bipolar Disorder Diagnostic Criteria

**DOI:** 10.3390/bs6030014

**Published:** 2016-07-15

**Authors:** Brittany L. Mason, E. Sherwood Brown, Paul E. Croarkin

**Affiliations:** 1 Department of Psychiatry, University of Texas Southwestern Medical Center; Dallas, TX 75390, USA; brittany.mason@utsouthwestern.edu; 2 Department of Psychiatry and Psychology, Mayo Clinic, Rochester, MN 55901, USA; croarkin.paul@mayo.edu

**Keywords:** bipolar disorder, manic depression, major depressive disorder, depression, mood disorder, diagnostic criteria, diagnostic and statistical manual of mental disorders, DSM, research domain criteria, history of bipolar disorder

## Abstract

Mood is the changing expression of emotion and can be described as a spectrum. The outermost ends of this spectrum highlight two states, the lowest low, melancholia, and the highest high, mania. These mood extremes have been documented repeatedly in human history, being first systematically described by Hippocrates. Nineteenth century contemporaries Falret and Baillarger described two forms of an extreme mood disorder, with the validity and accuracy of both debated. Regardless, the concept of a cycling mood disease was accepted before the end of the 19th century. Kraepelin then described “manic depressive insanity” and presented his description of a full spectrum of mood dysfunction which could be exhibited through single episodes of mania or depression or a complement of many episodes of each. It was this concept which was incorporated into the first DSM and carried out until DSM-III, in which the description of episodic mood dysfunction was used to build a diagnosis of bipolar disorder. Criticism of this approach is explored through discussion of the bipolar spectrum concept and some recent examinations of the clinical validity of these DSM diagnoses are presented. The concept of bipolar disorder in children is also explored.

## 1. Introduction

The overarching structure in which the changing expression of emotions is shaped is known as mood, and this long-term mood fluctuates over time [1]. Emotional expressions can be single experiences of intense emotion but also can be understood in the context of a longer-lasting emotional state over a period of time [2]. In a broad sense, mood can be described as a spectrum, describing how the various expressions of human happiness and sadness may be experienced. The outermost ends of this spectrum highlight two states which are linked to mental illness and are experienced in bipolar disorder; the lowest low, melancholia, and the highest high, mania. These mood states can also exist at the same time and overlap with the expression of emotions of conflicting affective states such as irritability with elation [1,2]. How these two states relate to each other and how mood shifts between states is explored in this review detailing the history of the bipolar disorder diagnosis.

## 2. Hippocrates: Melancholia and Mania

These two extremes of mood have been documented in human history as early as ancient Greek physicians and philosophers, and were first systematically described by Hippocrates (460–337 BCE) [3]. His work was based on both the contemporary anatomical explorations of Pythagoras, Alcmaeon, and Empedocles of Crotona and on his own clinical observations of these extreme expressions of mood [3]. First attempts to describe personality states used the hypothetical humors of the body, deriving choleric, phlegmatic, sanguine, and melancholic types [3] in an attempt to encompass all expressions of human emotion. “Melancholia” (“melas”, black, and “chole”, bile) described the pathological state of severe sadness [3] and mania was the result of an excess of yellow bile [4]. The presence of this mood state was apparent and described in the literature; however, the origin of the term “mania” is much less clear [3]. The Roman physician Caelius Aurelianus detailed at least seven possible etymologies and described two kinds of mania as defined by Plato, “one involving a mental strain that arises from a bodily cause of origin, the other divine or inspired” (translated by Drabkin, 1950, cited in [3]).

Work to define a mental disorder separate from a temperament started as early as Hippocrates, who distinguished between these two, defining the disease “melancholia” (nosos melancholiké) and the personality (typos melancholicós) [3]. Aretaeus also worked to emphasize the biological origin of melancholia, which differed from the psychological reaction termed “reactive depression” [3]. It was apparent to these men that the expressions of mania and melancholia were not innate personality traits, nor specific reactions to situations, but instead a biologically driven shift. Debate continued regarding how these two extremes may have been linked within one person and how these changing emotional states were expressed as a disease of the mind. Later work by Aretaeus of Cappadocia first linked these two states of mania and melancholia and expressed this concept of the spectrum, believing that melancholia and mania have the same etiology coming from brain dysfunction. Specifically, that mania is a worsening of melancholia and that mania is the phenomenological counterpart of melancholia [3]. During the mid-19th century, the concept of mania and melancholia and the link between these two states were re-examined and the “modern” bipolar disorder was formed.

## 3. Falret and Baillarger: A Cycling Disease

From antiquity through to the 19th century, mania and melancholia were considered to be two completely different disorders which embraced a wide variety of psychiatric syndromes. Jean-Pierre Falret created the first concept of a new and separate psychiatric disorder which encompassed both mania and depression and published his description of this disease in 1851, which he termed “folie circulaire”, a mental disorder characterized by a continuous cycle of depression, mania, and free intervals of varying lengths between these two extremes [3,5]. His contemporary Jules Baillarger described “folie à double forme” in which mania and melancholia change into one another but with no requirement for an free interval between the two, in contrast to Falret’s description which would include those with a long interval between the two mood states to still receive a diagnosis of “folie circulaire” [3]. These discrepant hypotheses regarding this aspect of cycling, with pauses between mood episodes still regarded as linked shifts between mood states regardless of length, created a rift in understanding when instead they may have been describing two sides of the same coin. Regardless, these competing descriptions spread from France via supporters of either description, and the concept of a cycling mood disease was accepted before the end of the century [3].

## 4. Kahlbaum and Kraepelin: A Comprehensive Description of Mood Dysfunction

Early classification of the psychoses into a nosological framework was first put forth by Karl Kahlbaum (1863), postulating a close correspondence between clinical symptoms, the disease course and outcome, brain pathology and etiology which suggested a natural disease entity [6]. Kahlbaum distinguished between two large groups of mental disorders on the basis of symptoms and outcome, one denoting a limited disturbance (“vecordia”) and the other denoting a complete disturbance of the mind (“vesania”) [5]. The first group presented with a continuous but remitting course whereas the latter presented with changing symptomatology and indicated a progressive disease. This distinction formed the framework on which numerous other signifiers were added, creating a complex, and ultimately unwieldy, classification system. Taking inspiration from both Kahlbaum and Falret, Emil Kraepelin finally tried to characterize all dysfunction of mood and to marry mania, depression and all psychotic states into what he termed “dementia praecox” and “manic-depressive insanity” [3,5], now known as schizophrenia and bipolar disorder, respectively. Work by Kahlbaum and Kraepelin was limited by research tools available at the time which prevented them from fully exploring and testing their hypotheses about the organic nature of these psychiatric illnesses. Although Kraepelin never fully detailed the diagnostic features of dementia praecox and manic-depressive psychosis, later works include rich clinical descriptions which allow for fairly accurate reconstruction of the psychopathological outlines of these two entities [6]. It is fair to surmise that manic-depressive insanity is a broader group than the current bipolar disorder and that dementia praecox is more restrictive group than the current schizophrenia. The Kraepelinian dichotomy of psychoses encompassed all possible disruptions of mood, and specifically in manic-depressive insanity, described the full spectrum of mood dysfunction which could be exhibited through single episodes of mania or depression or a complement of many episodes of each [7]. His description of manic-depressive insanity explains his position.

“Manic-depressive insanity…includes on the one hand the whole domain of so-called periodic and circular insanity, on the other hand simple mania, the greater part of the morbid states termed melancholia and also a not inconsiderable number of cases of amentia (confusional or delirious insanity). Lastly, we included here certain slight and slightest colourings of mood, some of them periodic, some of them continuously morbid, which on the one hand are to be regarded as the rudiment of more severe disorders, on the other hand pass without sharp boundaries into the domain of personal predisposition…I have become more and more convinced that all of the above-mentioned states only represent manifestations of a single morbid process”

(Kraepelin, translated and quoted in [7])

In this concept, all possible expressions of mood states exist in one categorical spectrum [7]. This would include together our modern definitions of depressive and manic states, psychosis, mixed states, and the subdromal expressions.

“…all the morbid forms brought together here as a clinical entity, not only pass over the one into the other without recognizable boundaries, but that they may even replace each other in one and the same case. On the one side…it is fundamentally and practically quite impossible to keep apart in any consistent way simple, periodic and circular cases; everywhere there are gradual transitions. But on the other side we see in the same patient not only mania and melancholia, but also states of the most profound confusion and perplexity, also well developed delusions, and lastly, the slightest fluctuations of mood alternating with each other. Moreover, permanent, one sided colourings of mood very commonly form the background on which fully developed circumscribed attacks of manic-depressive insanity develop”

(Kraepelin, translated and quoted in [7])

## 5. DSM-I, 1952: Manic Depressive Reactions

The first attempt to categorize and standardize mental illness in DSM-I (1952) classified manic-depression as a psychotic disorder, “characterized by a varying degree of personality integration and a failure to test and evaluate correctly external reality in various spheres” [8]. “Manic depressive reaction” details symptoms of mania true to the diagnosis today, severe mood swings and a tendency to remit and recur [8]. However, illusions, delusions and hallucinations are also listed as possible additions to the diagnosis and highlight the psychotic features now known to be only a small component of the wider disease. This diagnosis approaches more closely the Kraeplinian understanding of manic-depressive insanity, and the dominant mood presentation of a patient would be described using the specific type.

Three types were detailed: manic, depressed and other. The manic type details what is most similar to the modern definition of mania, “elation or irritability, with overtalkativeness, flight of ideas, and increased motor activity”, with depression only present in fleeting episodes [8]. The depressed type resembled what is now Major Depressive Disorder, “outstanding depression of mood and with mental and motor retardation and inhibition; in some cases there is much uneasiness and apprehension. Perplexity, stupor or agitation may be prominent symptoms” [8]. It is only in the other type in which mixed states or cycling is characterized as a feature, “marked mixtures of the cardinal manifestations of the above two phases (mixed type), or those cases where continuous alternation of the two phases occur (circular type)” [8]. These classifications allow for cases which do not easily fit into a primary manic or primary depressive description to be typed; however, the modern understanding of bipolar with a primary characteristic of cycling between mania and depression is not a fundamental component of the major disorder. In addition, the mixed state is mentioned but again was not fully characterized.

Schizophrenic reactions are a category synonymous with the earlier term dementia praecox, characterized by “fundamental disturbances in reality relationships and concept formations, with affective, behavioral, and intellectual disturbances in varying degrees and mixtures” (DSM-I) [8]. The schizo-affective type was “intended for those cases showing significant admixtures of schizophrenic and affective reactions. The mental content may be predominately schizophrenic, with pronounced elation or depression” (DSM-I) [8], although this description goes on to state that most cases eventually become most like schizophrenia.

## 6. DSM-II, 1968: Manic-Depressive Illness

With the advent of DSM-II, manic depression was characterized as “manic-depressive illness” and classified under Affective Disorders. The description detailed these disorders being characterized by a “single disorder of mood, either extreme depression or elation, that dominates the mental life of the patient” and that the onset of mood state is not precipitated by any specific life event [9]. Manic-depressive illness was now fully characterized with mood swings and the tendency to remit and recur and again separated into three types: manic, depressed, and circular. Manic type continued to detail the common features of mania and now specifies that “brief periods of depression sometimes occur, without true depressive episodes” [9] and is equivalent to the current concept of unipolar mania. For this description of depressed type, the depressed episodes were the only mood state present and again “uneasiness, apprehension, perplexity, and agitation may also be present” [9], as now described as unipolar depression. The circular type now encompasses the modern characteristic of cycling, “distinguished by at least one attack of both a depressive episode *and* a manic episode” and further sub-types into circular type, manic or circular type, depressed [9]. The mixed state was categorized as “Other major affective disorder” and clinicians are advised to place “major affective disorders for which a more specific diagnosis has not been made…[and] also for “mixed” manic-depressive illness, in which manic and depressive symptoms appear almost simultaneously” [9].

Schizophrenia was a separate disorder thereby still distinguishing thought disorder from those of affective illnesses, predominated by a disorder of mood. The schizo-affective type exhibited a “mixture of schizophrenic symptoms and pronounced elation or depression” and has the excited and depressed sub-types [9].

## 7. DSM-III, 1980: Manic Episode and Bipolar Disorder

The following edition of the DSM, DSM-III (1980), began to characterize illness with specific diagnostic criteria and shapes the modern definition of bipolar disorder, now termed as such. The biggest deviation from previous versions specified the symptom criteria required for the diagnosis of “episode” and it is the presence of varied episodes which will construct the final diagnosis. Another major divergence in this edition is the separation of unipolar and bipolar depression and a characterization that they are two different types of mood disorder. This emphasized the polarity of mood in this characterization, that the presence of any mania indicates bipolar disorder, rather than the episodicity of the aforementioned manic-depressive insanity/illness, in which the recurrence of the mood states was the primary characteristic [10].

Manic episodes and depressive episodes were detailed, as well as the possibility of a mixed episode. The ability to distinguish between these episodes and any schizophrenic-related diagnoses was commented on, mentioning that delusions and hallucinations may be present and would then receive a “with psychotic features” specifier on the episode in question. It was suggested that if distinguishing between presenting symptoms was particularly difficult, the clinician should use additional complementary criteria to help form the diagnosis, for example, using a family history of affective disorders or a previous affective disorder episode to give an Affective Disorder diagnosis rather than a Schizophrenic-related one, but that the Schizoaffective diagnosis would be appropriate if the clinician was not confident in distinguishing one from the other. Diagnostic criteria for a manic episode included: an increase in activity (either socially, at work, or sexually) or physical restlessness; more talkative than usual or pressure to keep talking; flight of ideas or subjective experience that thoughts are racing; inflated self-esteem (grandiosity, which may be delusional); decreased need for sleep; distractibility, i.e., attention is too easily drawn to unimportant to irrelevant stimuli; excessive involvement in activities that have high potential for painful consequences which is not recognized, e.g., buying sprees, sexual indiscretions, foolish business investments, reckless driving [11]. Importantly, at least three of these symptoms needed a duration of one week, for most of the time, though an episode would meet criteria regardless of duration if any hospitalization is required, unless only irritable mood was present and then four symptoms were required. The criteria further detailed symptoms which would preclude a manic episode diagnosis and points to other more appropriate diagnoses if schizophrenic symptoms are present (i.e., hallucinations or bizarre behavior), and as described above, guides the clinician in appropriately ascribing a “with psychotic features” specifier to the episode or giving a non-Affective Disorder diagnosis where appropriate.

The description for a manic episode more fully illustrates previously highlighted symptoms, “elation or irritability, with overtalkativeness, flight of ideas, and increased motor activity” [8]. Elevated mood is described as “euphoric, unusually good, cheerful or high; often has an infectious quality for the uninvolved observer; but was recognized as excessive by those who know the individual well” and is considered the “prototypical symptom”, mentioning that irritability may be present or the predominant mood symptom, likely when the individual is “thwarted” [11]. Overtalkativeness was explained as “manic speech [being] typically loud, rapid, and difficult to interrupt” [11] and flight of ideas as a “nearly continuous flow of accelerated speech with abrupt changes from topic to topic” [11]. Hyperactivity encompasses increased activity in numerous social domains and “often involves excessive planning of and participation in multiple activities...almost invariably there is increased sociability...the intrusive, domineering, and demanding nature of these interactions is not recognized by the individual” [11], but the lack of need for sleep was specifically mentioned, “awakens several hours before usual time, full of energy...may go days without any sleep at all and yet not feel tired” [11]. “Frequently, expansiveness, unwarranted optimism, grandiosity, and lack of judgement lead to such activities as buying sprees, reckless driving…have a disorganized, flamboyant, or bizarre quality” [11].

Lability of mood is detailed in which “rapid shifts to anger or depression” are present and “may last moments, hours, or, more rarely, days. Occasionally the depressive and manic symptoms intermingle” [11]. If the depressive symptoms are more prominent and last at least one full day, this would be best categorized in a Mixed episode, now defined as a separate mood state as opposed to an “other” category [11]. Psychosis, manifested through delusions or hallucinations, is not a core component but suggested that it may be present in some patients with the content normally consistent with the predominant mood (mood-congruent), but can also be present in a mood-incongruent fashion, though this is less common [11]. Apparently, the utility of congruent and incongruent characterizations was debated at length as a note to this point is included in the diagnostic criteria. 

The term “hypomania” was introduced and to be “used to describe a clinical syndrome that is similar to, but not as severe as” mania [11]. Atypical bipolar disorder was created to allow for those individuals who do not fit into the Bipolar or Cyclothymic Disorder diagnoses. It is mentioned as an example someone who is presenting with hypomania and first uses the term “Bipolar II” [11]; however this is only in the context of the atypical diagnosis and does not become its own diagnosis class until a later version of the DSM. The presence of hypomanic periods in the absence of a full manic episode would constitute a diagnosis of Cyclothymic Disorder [11]. Interestingly, Bipolar Disorder diagnosis requires the manic episode but not the depressive episode(s) to be present; however, the Cyclothymic Disorder diagnosis requires both mood disturbances to be present.

The sub-type of schizo-affective type of schizophrenia becomes Schizoaffective Disorder, though no specific criteria were presented. The clinical guidance details how many cases previously diagnosed with the schizo-affective type would now better fit into new diagnoses, such as Schizophreniform Disorder or Bipolar Disorder with Mood-congruent or Mood-incongruent Psychotic Features, and allowed the clinician freedom to use their judgement to use this new diagnosis when other more specifically defined diagnoses to not seem to fit the clinical presentation [11]. Also, if the manic episode was linked to a separate etiological cause (i.e., drugs or disease), it would not be diagnosed as a manic episode.

## 8. DSM-III-R (1987): Bipolar Disorder and Hypomanic Syndrome

The revised version of DSM-III clarified the description from “physical restlessness” to “psychomotor agitation” as a component of the goal-directed activity criteria for a manic episode [12]. A “Hypomanic Syndrome” would be diagnosed if the symptom criteria are present but the marked impairment component is not sufficiently met [12], resulting in a less severe diagnosis.

## 9. DSM-IV, 1994: Bipolar Disorder and a Mixed Episode

The DSM-IV (1994) diagnosis of a Manic Episode remained the same as the DSM-III/DSM-III-R version. Manic symptoms should not meet criteria for a Mixed Episode, now officially introduced as its own episode with diagnostic criteria, and the mood disturbance must be sufficiently severe enough to cause “marked impairment in occupational functioning or in usual social activities or relationships with others” [13]. A Mixed episode would be diagnosed if criteria for *both* a Manic Episode and a Major Depressive Episode (MDE) are met (excluding duration) for nearly every day for at least one week [13]. Hypomania can now be diagnosed episodically, the Hypomanic Episode having the same symptom criteria as a Manic Episode but instead now needing to last only 4 days and being clearly different from the non-depressed mood [13]. It does not require marked impairment in functioning but must also be free from psychotic features [13]. For all these episode diagnoses, episodes which are caused by somatic antidepressant treatment do not to count toward a Bipolar I diagnosis [13]. No changes to these diagnostic criteria were seen in DSM-IV-TR, nor was any text revised for these specific episodes [14].

## 10. DSM-5, 2013

There was some departure from the previous DSM in this edition that may satisfy some of the issues with the diagnostic criteria which are discussed later in this review. The criteria for all episodes (manic, hypomanic and depressed) remained the generally same with a minor but important adjustment. The mood criteria for a manic episode now require both the mood disturbance and increased goal-directed activity/energy, rather than the increased energy to be another symptom which is present. However, now if a manic episode emerges during antidepressant treatment but persists at a syndromal level well past that of a treatment effect, this would now meet criteria for a manic episode and therefore a Bipolar I diagnosis [15]. This is also mentioned for a Hypomanic Episode, but instead specifies that the presence of one or two symptoms following antidepressant treatment does not constitute a hypomanic episode nor directly indicate a bipolar disorder [15]. Importantly, mania which is induced by substance or medication is now classified as a bipolar disorder, but one that is specific to this precipitating factor [15]. This was a deviation from the DSM-IV in which substance-induced mood disorder was the only possible category for this condition and would not be classified as bipolar. In addition, the Mixed Episode was removed as a separate diagnostic category and converted into the “with mixed features” specifier which can apply to either a manic or hypomanic episode with depressive features (dysphoria or depressed mood, anhedonia, psychomotor retardation, fatigue, guilt or worthlessness, suicidality) or a depressive episode with manic features (elevated mood, grandiosity, more talkative than normal, racing thoughts, increase in goal-directed activity, increased risky behaviors, decreased need for sleep), and it was noted that mixed features which are associated with a major depressive episode have been shown as a significant risk factor for the development of Bipolar I (BP I) or II (BP II) and therefore pose an important clinical distinction to guide treatment [15]. An important distinction may be indicated by the reorganization of chapter classification, with bipolar disorder and depression being moved to separate chapters in DSM-5, lending more weight to the idea that these two disorders are truly separate diseases. 

Confusingly, the inclusion of a mixed features specifier for both depression and mania emphasizes the similarity between the possible symptoms and the clinical experience that expressions of mania and depression are frequently not so clear. This mixed features signifier for DSM-5 allows for characterization of mixed features in both an MDE and a manic/hypomanic episode. Individuals participating in a multi-site, naturalistic, cross sectional study encompassing both outpatient and inpatients, were evaluated [16]. A total of 982 patients with a current diagnosis of Major Depressive Disorder (MDD), BD-I or BD-II, a current MDE or hypo/manic episode were included and were then characterized by a Young Mania Rating Scale (YMRS) and/or a Montgomery Åsberg Depression Rating Scale (MADRS) for a mixed features signifier to be assigned post-hoc [16]. A significant percentage of individuals (i.e., approximately 25–35%) fulfilled DSM-5 criteria for mixed features and individuals who met criteria for mixed features exhibited a more severe depressive phenotype as evidenced by greater scores on the MADRS and Hamilton Rating Scale for Depression (HAMD)-17 item. Individuals with mixed features also exhibited a higher rate of alcohol/substance use disorder in the context of Bipolar Disorder but not MDD [16]. These data support the hypothesis that the presence of manic symptoms indicates a different, and likely more complex and severe, disease course.

## 11. Evaluating Diagnosis Nosology

Modern biological research techniques, including neuroimaging and genetics, provide new insight into the causes of mental illness; however, they also add data to challenge the validity of the nosology of the psychoses and other psychiatric disease which are in current practice today. Few significant genome-wide linkages for currently diagnosed phenotypes have been found but many low-order positive lod scores have been detected for both schizophrenia and affective disorders, and neuroimaging studies have found many overlapping features [6]. These conflicting data highlight the clinical experience that many patients to do not fit neatly in the current diagnostic framework. Originally in DSM-I, Bipolar Disorder was considered a psychotic disorder but then classified from DSM II on a mood disorder, and later editions of the DSM seem to move further and further from Kraepelin’s original characterization of the clinical presentations which he studied. Modern analysis of Kraepelin’s clinical documents and disorder classifications using the original clinical features and a modern classification algorithm which grouped these patients into diagnostic classes corresponding to ICD-9 categories revealed strong “goodness of fit” within Kraepelin’s typology of the psychoses and the clinical material on which it was based [6]. However, this approach is only one which has been proposed and its validity has been questioned. 

Kraepelin’s framework for the psychoses was challenged strongly at the time when it was first proposed. Hoche (1912) disagreed with the assumption of a linear relationship between localized brain dysfunction, whether lesion or microchemical, and the clinical symptoms of psychotic illness [5,6]. He felt that psychopathology should be limited to achieving exact descriptions of symptom-complexes which were not tied to any specific etiology. Similarly, Bonhoeffer (1912) pointed out that the same etiology can result in widely different clinical manifestations and that the complex nature of psychological symptoms prevented them from being differentiated any more specifically than clusters of “reaction types” [5,6]. Wernicke (1906) used a neurological understanding to hypothesize three functional brain systems involving the association cortex which go awry in psychoses: psychomotor, psychosensory, and intrapsychic, and various disturbances would then lead to psychotic syndromes [6]. Kleist (1953) challenged the Kraepelinian model with a distinction between bipolar and monopolar psychoses, mania and depression being separate diseases and bipolar psychosis being a particular affiliation of both [5] and this work with contributions by Leonhard (1979) formulated a complex classification system of the psychoses in which schizophrenia could be caused by genetics (unsystematic form) or environment (systematic form) [6]. This system proved difficult to use clinically and has not been maintained. Kraepelin himself called into question his own framework which was utilized to shape understanding of psychoses, “it is natural to turn away from arranging illnesses in orderly well-defined groups and to set ourselves instead the undoubtedly higher and more satisfying goal of understanding their structure…we cannot distinguish satisfactorily between these two illnesses and this brings home the suspicion that our formulation of the problem may be incorrect” [6].

Within this diagnostic framework, the question remained of where to categorize those patients who presented with schizophrenic symptoms as well as a psychotic mood disorder. The addition of this schizoaffective diagnosis attempted to categorize this group; however the usefulness of this diagnosis has been questioned [17]. A potential point of confusion for those using DSM is distinguishing between schizoaffective disorder (psychosis between mood episodes) and a mood episode that includes psychotic features (bipolar I disorder with a psychotic features specifier). A prospective examination of 413 patients beginning at first admission for psychosis, followed over a 10-year period, attempted to clarify where the boundary between schizophrenia and psychotic mood disorders may lie [17]. Clinical diagnoses were grouped into schizophrenia/schizophreniform, schizoaffective, bipolar with psychosis, depression with psychosis, and other psychoses and outcomes were assessed using the Global Assessment of Symptoms (GAS), the Global Assessment of Functional Performance (GAF-F) and the Global Assessment of Functioning (GAF) for the best month between the 4th and 10th year [17]. Statistical modeling and multiple regression analyses were used to correlate symptom course variables and outcome measures and revealed a qualitative difference between cases in which psychosis is limited to the mood episode and those cases in which the psychosis is nonaffective and did not support the existence of a distinct schizoaffective disorder [17]. Mania was seen to be either episodic or chronic and this coupled with the finding related to psychosis led to the conclusion that the clinical picture was better represented in the Kraepelinian dichotomy of manic-depressive illness as opposed to the DSM continuum description [17]. Recent studies have highlighted the common genetic factors between schizophrenia and bipolar disorder and the shared features of bipolar disorder with unipolar major depression, psychotic depression, schizoaffective disorders and other psychotic disorders lead some to suggest that it lies at a genuine interface between affective and psychotic disorders [18]. The current clinical concept of bipolar disorder is based in the concept of polarity, in which the expressions of mania and depression are two ends of a single mood dimension; however, many have noted that increased activation is a core component of mania whereas decreased activation is present in some but not all depressed states, thus the “bipolar” dimension is one of motor and psychic activation and not one of mood. Instead, if researchers could use a multi-axial system which encompasses all states of mood dysfunction, a continuous unidimensional measure of depression severity and psychotic symptoms, a bidirectional measure of motor and psychic activation, the path to finding the mechanistic neurobiological underpinnings of where this “switch” between activation states (low to high or vis-a-versa) may be, as well as linking in shifts in circadian rhythms and eating behaviors, may become more clear [18].

However, the question of where to place psychotic symptoms within the framework of clinical diagnosis is still confusing. Meyer and Meyer (2009) sought to examine this issue using identical vignettes of a clinical presentation adjusted only for the presence of auditory hallucinations (more commonly associated with psychotic disorders) and the decreased need for sleep (one possible symptom of mania) to evaluate how actual psychiatrists would diagnosis and what subsequent treatment they would recommend. Although the vignette was written to clearly present a patient with mania warranting a diagnosis of Bipolar Disorder (DSM-IV criteria), almost half of the psychiatrists gave an inappropriate diagnosis (45% total; 41.5% psychotic disorder, 3.5% other diagnosis), with a manic episode diagnosed by 37.3% of the psychiatrists and bipolar affective disorder diagnosed by 16.9% of the psychiatrists [19]. Recommended treatment did not diverge from appropriate treatment for the bipolar diagnosis but it is mentioned that other studies found a negative treatment impact regarding an inappropriate diagnosis [19]. Examining this question of “accurate” diagnosis with regards to genetic linkage studies, Roy et al. (1997) used sophisticated statistical analyses to assess where the issues with diagnoses may lie. They found that when both affective and psychotic features were present, disagreements between diagnoses was more likely, compared between clinicians blinded or unblinded to a familial diagnostic lineage [20]. In addition, the diagnosis of schizoaffective disorder (DSM-III-R criteria) showed the worst confusability coefficients, this diagnosis being frequently disagreed with schizophrenia, schizophreniform disorder, bipolar I disorder as more appropriate diagnoses [20]. The multisite Bipolar-Schizophrenia Network on Intermediate Phenotypes (B-SNIP) study analyzed biomarkers (e.g., neuroimaging) in psychotic patients with schizophrenia, schizoaffective disorder and bipolar disorder [21]. An analysis of biomarkers from the participants in this study revealed three biotypes that cut across the diagnostic boundaries while the use of DSM criteria was more consistent with a continuum of severity from schizophrenia to bipolar disorder. These findings suggest that the inclusion of biological data can lead to quite different classification of a symptom domain such as psychosis than using conventional diagnostic criteria.

## 12. Unipolar Mania

The singular manic state, without the pairing of depression, was a possible diagnosis from DSM-I (1952). It is mentioned that depression could be present but that these periods would be brief experiences. From DSM-III (1980), the manic state becomes a diagnosable manic episode and becomes sufficient to receive a diagnosis of Bipolar Disorder, though it is mentioned in the text that “most individuals who have a disorder characterized by one or more manic episodes (Bipolar Disorder) will eventually have a major depressive episode…[and] in Bipolar Disorder the initial episode is often manic. Both the manic and the major depressive episodes are more frequent and shorter than the major depressive episodes in Major Depression. Frequently a manic or major depressive episode is immediately followed by a short episode of the other kind. In rare cases, over long periods of time, there is an alternation of the two kinds of episodes without an intervening period of normal mood (cycling)” [11]. Thus, the understanding at this time is that mania would not exist (in a longitudinal sense) without depression and therefore mania is an indication of the larger condition of Bipolar Disorder. Data and hypotheses detailed below contradict the idea that mania cannot present without an eventual depressive episode.

## 13. A Bipolar Spectrum

The defining of two states of depression, unipolar in the absence of mania, and bipolar, in the presence of mania, has been criticized since its inception in DSM-III and some have desired to return to an earlier description of mood dysfunction [10]. It is suggested that these two forms of depression are not the same, but that they are distinctly different. Using a large, representative community sample (34,653), the specific symptoms of an MDE were mapped and compared between those with a history of manic symptoms and those without a lifetime history of manic symptoms [22]. These symptoms were assessed broadly, asking if someone endorsed at least one instance of a one week period in their life in which they felt “so extremely excited, elated, or hyper that you weren’t your normal self, that other people were concerned about you, or were so irritable or easily annoyed that you would shout at people or throw or break things?” [22], and detected a lifetime prevalence rate of 18.7% in this sample. This comparison revealed substantial differences in symptom expression in those with unipolar symptom group compared to a bipolar spectrum symptom group [22]. Sadness, appetite disturbance, and psychomotor symptoms were much better indicators of a depression severity in individuals without a lifetime history of manic symptoms compared to those with a history [22]. These data again highlight that the presence of manic symptoms indicates a distinctly different disorder, with its own expression of symptoms and likely course and outcome.

The categorical approach of the DSM-III and assumes that there are clear boundaries between disorders and that the extremes of mood exist in polarity. This is in contrast to the Kraepelinian understanding in which all mood dysfunction exists in a spectrum and the existence of mixed episodes is evidence which supports these ideas (e.g., if mood was truly of a polar fashion, symptoms of mania and depression could not exist together in time). The spectrum view of mood disorders takes a dimensional approach in which the types of mood dysfunction exist on a continuum and the severity is graded per the associated symptoms [23]. The concept of a bipolar spectrum harkens back to Kraepelin’s original description of mood dysfunction as a spectrum, in which repeated mood episodes, regardless of whether they are episodes of mania or depression, would reflect a specific type of disorder. Hagop Askiskal details this concept and proposed that an expansion of the diagnostic criteria for bipolar would better capture the vicissitudes that are seen in clinical presentation [24]. Using his characterizations, the spectrum of bipolar illness encompassed broader manifestations of bipolar illness: Bipolar I described the full-blown manic episode which occurs in the context of more regular depressive episodes, Bipolar I ½ described protracted hypomania in the context of depression, Bipolar II described depression with definite hypomanic episodes, Bipolar II ½ described cyclothymic depression with shorter hypomanic episodes, Bipolar III described hypomania induced by antidepressant treatment or the cessation of it, Bipolar III ½ described mania which can be induced by substance abuse, and Bipolar IV would be a lifelong hyperthymic temperament with clinical depression [24]. Under these broader descriptions of bipolar disorders, it is estimated that 4%–5% of the general population would qualify for a diagnosis, an increase from the commonly cited 1% of the general population under DSM criteria [25]. The currently used 4-day criterion to diagnose a hypomanic episode has also been challenged as arbitrary and thus allowing for those with manic symptoms of a shorter duration to slip through the cracks [26]. These patients may present a more complex phenotype and be yet another clinical example of another disease state which exists on this bipolar spectrum [26].

## 14. The Mixed State

A fundamental question to the puzzle of bipolar is the concept of the “state”. The argument between Falret and Baillarger concerning the interval, whether or not the space in between episodes was a fundamental component of the disease, still haunts the understanding of bipolar disorder. It is apparent that mania is a core symptom which may construct itself as an “episode”, as is the presence of depression; however, how discrete must these two mood states be to be considered two episodes? Recent explorations of the clinical presentations and longitudinal course of those with bipolar disease or mood dysfunction contributes data to help clarify the operational definitions of these disorders. The EPideminology of MANia (EPIMAN)-II Mille study collected data from 19 medical centers across France and examined the disease course for 981 patients, 73.6% being diagnosed with bipolar I with free intervals and 26.4% being diagnosed as bipolar I without free intervals [27]. The prevalence rate for those patients without free intervals was similar to that reported in the DSM-IV describing the persistence of residual symptoms following a mood episode, and those without free intervals were more likely to be single or divorced, have an earlier age of onset, and longer delay to receive first mood stabilizer treatment [27]. Although this study examined only inpatients in a retrospective fashion using DSM-IV criteria and were limited in the manner to assess interepisode recovery, the authors suggest they have identified both forms of bipolar described by Baillarger and Falret, being early and later onset bipolar disorder, respectively [27].

When examining aspects of the depressive state, the forms of agitated and psychotic depression do not match cleanly with the nosological grouping with depressive states [28]. They also do not correspond to antidepressant treatment response, which counterintuitively actually increase or induce agitation, delusions, insomnia and suicidal acts [28]. Agitated melancholia and agitated depression were once considered mixed affective states but this has not been continued in current versions of the DSM [28]. Numerous historical classifications of melancholic states from the 18^th^ and 19^th^ centuries have described an excited-agitated form of depression. Kraepelin described depressive or anxious mania in which flight of ideas frequently exhibited in speech was a key component, as were a great restlessness and an anxiously despairing mood, and this description is closely related to the previous agitated melancholia (melancholia agitata) [28]. Another form of the mixed state was described with excited depression, characterized by inhibition of thought, great restlessness, and anxious and despondent mood, differing from the previous form by an inhibition of the flight of ideas, with even a third state possible, depression with flight of ideas [28]. Over time, these specific diagnostic categories have been subsumed into depression and this has allowed the concept of a disease, which is melancholia, to become a syndrome of agitated depression, and then a single symptom, agitation, of a depressive episode. It is proposed that in order to make a diagnosis of agitated depression, in addition to major depression and inner agitation, at least three additional symptoms must be present: (1) racing or crowded thoughts; (2) irritability or unprovoked feelings of rage; (3) absence of signs of retardation; (4) talkativeness; (5) dramatic feelings of suffering or frequent spells of weeping; (6) mood lability and marked emotional reactivity; and (7) early insomnia [28]. Appropriate treatment would be achieved through antipsychotics, benzodiazepines, anticonvulsants, or lithium [28].

The question of the mixed state presents an even more complex picture of the bipolar course. Within a mixed state, both symptoms of mania and depression are present within a similar/exact timeframe, thus the mixed state highlights the similarity of depression and mania. Both can have agitation, cognitive changes and insomnia and indicating rather than opposite extremes of mood they may be more akin to two sides of the same coin. Whether or not the mixed state even exists as a standalone episode is debated, believed by some instead to be a transitional phase between manic and depressed episodes [29]. Recognition of the mixed state became codified in the DSM-III, though the possibility of a mixed state was detailed from the first inception of the DSM and spoken of by Kraepelin. One explanation of the mixed state describes it as a transitional period between mood states; however, it is argued that the mixed state may represent a truly distinct mood state which is composed of symptoms of both depression and hypomania and can be linked to particular characteristics [25]. Plotting the distribution of co-occurring hypomanic symptoms between bipolar II depression and major depressive disorder as a histogram shows a normal distribution [23]. Specific depressive symptoms do seem to cluster, with those of bipolar depression more likely to involve hypersomnia and psychomotor retardation whereas major depressive disorder has been reported to more likely involve insomnia and psychomotor agitation [23], and as detailed above, sadness, appetite disturbance, and psychomotor symptoms are shown to be much better indicators of a depression severity in individuals without a lifetime history of manic symptoms compared to those with a history of manic symptoms [22]. Bipolar II has been found to involve more atypical symptoms (hypersomnia, overeating) and more co-occurring hypomanic symptoms (including psychomotor agitation) compared to major depressive disorder. Mixed depression, as defined by a combination of depression and manic/hypomanic symptoms not including elevated mood, is not classified in the DSM (IV-TR) but has been described in Bipolar I, II, and MDD; however, it does follow historical descriptions by Falret, Kraepelin, and others [23].

Outpatients presenting with aMDE with 348 having a BP II diagnosis and 254 having a MDD diagnosis were interviewed (SCID DSM-IV, structured Family History Screen, and Hypomania Interview Guide) and examined for atypical features which would receive the “atypical features” specifier per DSM-IV [30]. Depressive mixed state was defined as greater than or equal to 3 concurrent hypomanic symptoms during the MDE. Atypical depression was present in 43% of the combined BP II and MDD sample, when comparing AD vs. non-AD, the AD group had significantly higher rates of BP II and was significantly associated with all bipolar validators, the most robust being family history (dose-dependent relationship between bipolar family history loading and number of atypical symptoms-most being leaden paralysis and hypersomia) [30]. These data suggest that atypical depression should be viewed as a variant of BP II and that when presented with an MDE with atypical features, a BP II diagnosis should strongly be considered, and that this form of depression could serve as a nosological bridge between unipolar depression and bipolar disorder (through BP II). When using a definition of mixed depression which includes three or more co-occurring hypomanic symptoms, the presence of mixed depression shows high positive predictive value for Bipolar II disorder [23].

This distinction has important clinical implications regarding achieving appropriate pharmacological treatment. The use of antidepressants in patients with bipolar disorder is not fully supported by the data [31]. The data do indicate that some individual patients may benefit from antidepressants but that there is a possible risk for a switch to mania/hypomania or a mixed state and therefore, patients with bipolar disorder should be treated in combination with a mood stabilizer as opposed to antidepressant monotherapy [31]. Particular concern should be paid to the use of tri- and tetracyclics and venlafaxine as there does seem to be high risk for induction of pathologically elevated mood and behavior [31]. Antidepressants alone in a mixed state or in Bipolar II may increase the severity of irritability and psychomotor agitation, reported to be possible precursors to suicidality [23]. Importantly, the STEP-BP study indicated that adjunctive treatment of antidepressants with mood stabilizers was not associated with greater efficacy nor a greater risk for an affective switch [32] and that adjunctive treatment with lamotrigine, inositol, or risperidone to antidepressant treatment provided no significant improvement in depression for bipolar depression [33]. Only lamotrigine improved the recovery rate and was associated with lower depression severity when compared to the other treatments [33]. A reanalysis of the STEP-BP study revealed that subjects receiving low-dose antidepressants were less likely to achieve durable recovery when compared to those on higher doses; however, the recovery rates for those on high-dose antidepressants was not significantly different from placebo [34]. There was no significant difference in the rate of treatment-emergent affective switching between groups, rather that switching was more likely if the subjects had been diagnosed with BP I or presented with concomitant manic symptoms at baseline [34], again highlighting this very important distinction between those which display mania at any point compared to those who exhibit true unipolar depression.

A review of those studies which used the Mood Spectrum Structured Interviews (SCI-MOODS) or the self-report version (MOODS-SR), an assessment designed to collect the life-long sub-threshold mood dysregulation present surrounding both manic and depressive episodes, attempted to clarify the mood spectrum model [35]. This assessment was designed in part to recognize sub-diagnostic and atypical features of mania and hypomania in both bipolar and unipolar patients, and successfully identified mild hypomanic features among 117 patients diagnosed with remitted recurrent unipolar depression and 106 patients with bipolar I disorder (both DSM-IV diagnoses) [35]. This assessment can best characterize the “mixed state”. In addition, these findings highlight the intrinsic issue with the concept of “pure” unipolar depression when the clinician does not have longitudinal data to continually confirm the original diagnosis, or well before any episode may meet diagnostic criteria to warrant an adjustment of diagnosis. Reporting higher scores on the MOODS-SR factors which assessed psychomotor disturbances, mixed instability and suicidality delineated those patients who also experienced a more severe form of disease, a higher risk for psychotic symptoms, and a lower quality of life after remission [35]. The authors argue for the mood spectrum approach, suggesting a continuum from “pure mania” to “pure depression” and no true demarcation between the two realms, to best describe the expression of human mood, and shaping clinical understanding of dysfunction of the expression of mood [35]. In DSM-5, the mixed state has been replaced with a “mixed features” specifier than can be used for either depressed or manic mood states. The impact of this change on diagnoses in clinical practice is, to date, unclear. This change could decrease the use of a bipolar disorder diagnosis by giving clinicians the option of a depression diagnosis as is part of MDD. Alternatively, it could lead to more consistent diagnosis by allowing for diagnosis of sub-syndromal mixed states or result in confusion regarding the boundary between bipolar disorder and unipolar depression.

Genetic studies have also examined the biology which may influence disease processes. Genetic association of 447 probands and 2082 first-degree relatives found familiar aggregation of bipolar disorder and major depression, suggesting distinct underlying pathways rather than increasingly severe manifestations of a shared diathesis [36]. Heritability was greatest for bipolar I (primarily due to the presence of mania; 0.83, s.e. = 0.08, P = 1.69E–26, OR = 8.27, 3.82–17.91) and MDD also showed significant association (0.54, s.e. = 0.05, P = 1.38E–42, OR = 2.45, 1.66–3.62) [36]. These results support the hypothesis that mania and depression are actually distinct disease processes which occur co-morbidly in bipolar disorder. It is also suggested that the low reported incidence of unipolar mania may be an artifact of clinical sampling rather than a true rarity, and highlights the importance of using symptom-specific diagnostics which cross the current diagnostic categories.

## 15. Historical and Contemporary Conceptualization of Bipolar Disorder in Children and Adolescents

Diagnostic criteria and prevalence rates for bipolar disorder in children and adolescents are controversial. The ambiguity of this diagnosis in development has its roots in quandaries focused on to what extent bipolar disorder is a spectrum with a range of symptoms on the border of psychosis to normal behaviors, characterizing episodes of depression with fluctuations in mood that reliably identify periods of hypomania or mania, differentiating mood episodes from longstanding temperamental traits, differentiating primary symptoms from the effects of psychotropic medications or substances of abuse, developmental shifts in phenotypic symptom presentations, and differentiating mood disorders from other childhood disorders [37]. These diagnostic predicaments have been pondered for almost a century [37,38,39].

Nonspecific descriptions of children with putative mania date back to the 18th century [37,40]. In the 1850s, two researchers attempted to unify the concept of manic depression. As described above, Jean-Pierre Falret described a disorder of fluctuating cycles of depression, mania, and euthymia that he called “folie circulaire” and Jules Baillarger, a local colleague of Falret’s developed the diagnosis of “folie a double forme” which had periods of mania and melancholia morphing in to one another with inconsequential inter-episode intervals [37]. Ultimately, these two concepts were combined and in 1898 a lucid case history of a 13-year-old with “folie circulaire” was presented. This early description is congruent with the contemporary diagnostic construct of bipolar I [41]. Kraepelin also discussed the prospect of manic depression presenting in childhood [42,43]. Theodor Ziehen was a German physician who wrote prolifically on psychiatric disorders in childhood. Ziehen characterized childhood mania as a distinct entity on a spectrum with normal development, emphasized the recognition that manic symptoms represent a sudden shift from normal behavior, and commented on the potential inherent challenges in differentiating mania from normal development [44]. Interest in childhood manic-depression continued thereafter with systematic studies often characterizing postpubertal, depressive presentations [45,46,47]. Charles Bradley, widely considered the father of experimental child and adolescent psychopharmacology reportedly felt that mania was rare in children and discouraged use of the diagnostic construct in this population. However, Bradley’s serendipitous work in the 1930s with Benzedrine sulfate for youth with behavioral disorders may have included participants with mood disorders [48].

Of note, other early constructs of bipolar disorder were derived from psychoanalytic theory. Karl Abraham, Melanie Klein, and Adolf Meyer all commented on manic-depressive symptoms in childhood [37,43]. Abraham linked mania with oral stage but emphasized the contributions of physiologic and psychologic factors in symptom presentations [49]. Klein described a transient, manic-depressive, developmental phase as a part of childhood with hypomanic and manic defenses. Further she believed symptomatic manic-depressive patients failed to develop healthy, infantile object relations and successfully navigate the “depressive position” during the first year of life [50]. Current experts have also related that diagnostic criteria for manic-depression in children were likely underdeveloped initially as early, prominent psychoanalytic scholars felt that children lacked essential cognitive structures that materialize after pubertal psychosexual stages. Early child psychoanalysts were also potentially treating a healthier population of patients as compared to hospitalized children [37].

Historically, descriptive criteria for bipolar disorder in children and adolescents trailed the development of adult criteria. Disorders of childhood and adolescence were first characterized in DSM-II. Two pages of DSM-II were devoted to this area and included broad constructs such as hyperkinetic reaction of childhood [9]. Weinberg and Brumback are often credited with developing modern, descriptive criteria for mania in children [43], and advocated for the adaptation of this concept despite the controversy. Of note, patients in these early case reports were often characterized as hyperactive, among other constellations of symptoms [51]. These early efforts galvanized structured and semi-structured interviews as well as more symptom-driven approach for characterizing psychiatric illnesses in children and adolescents. In 1980, DSM-III presented more fully developed criteria for child and adolescent disorders. Notably and foreshadowing future controversies, associated features of attention deficit disorder with hyperactivity included “negativism” and “increased mood lability” [11]. With DSM-IV [13] and DSM-IV-TR [14] descriptive criteria in general were refined to include manic, mixed, and hypomanic episodes. Recently, DSM-5 brought pertinent changes in descriptive criteria for affective disorders [15]. Arguably these changes have elicited considerable controversy in the diagnosis and treatment of youth and for the first time in the history of DSM criteria, developmental perspectives have been given sustained consideration. Specifically, this includes the mixed features specifier that can be applied to a manic or depressive episode. This change has great utility for the diagnosis and treatment of children with mood disorders as mixed symptom presentations are common and concerning in children and adolescents [52]. Further, disruptive mood dysregulation disorder (DMDD), a new depressive disorder diagnosis was introduced. A central, impairing diagnostic criteria for DMDD includes severe, long standing irritability punctuated by pathologic temper tantrums. The genesis of DMDD was driven by prior research efforts focused on severe mood dysregulation and concern over an accelerating, contemporary trend in which children and adolescents with noncyclical irritability have been diagnosed with bipolar disorder [53].

There has been considerable skepticism among clinicians and researchers regarding the DMDD diagnostic construct. Leibenluft and colleagues originally characterized a “severe mood dysregulation” phenotype in adolescents with hyperarousal, chronic irritability, and increased affective instability [53,54]. Research suggests that youth with severe mood dysregulation vary from those with bipolar disorder with respect to family history [55], long-term outcomes [56], and neurobiology [57]. The DMDD diagnosis was modified slightly from severe mood dysregulation in that hyperarousal is not included, and if more than 1 day of hypomanic or manic symptoms are present, the DMDD diagnosis is not applied. Opponents of the DMDD diagnostic construct maintain that empirical support for this dramatic change in criteria is lacking, and furthermore, no definitive information on the prevalence of DMDD currently exists. In addition, the stability of the diagnosis is questionable in some reports. Existing information suggests that DMDD is highly comorbid with other mood and disruptive behavior disorders, thus also bringing it validity into question [53]. Studies of outpatient DMDD samples also indicate suboptimal reliability [58]. Psychosocial interventions, stimulant medications, mood stabilizers, antidepressants, and antipsychotics have been pondered as potential interventions. Detractors also have raised concerns that there are no effective, evidence based treatments for DMDD [59,60] and that clinical guidelines are based on extrapolation from other mood or disruptive behavioral disorders. Presently, it is unclear if the DMDD diagnostic construct will have clinical utility, reduce the rate of bipolar diagnoses in childhood, or improve clinical outcomes for families [53].

Proponents of DMDD suggest that this construct allows clinicians to diagnosis and treat impairing symptoms in youth without imparting the idea that these patients will have a lifelong disease such as bipolar disorder. Conversely, two large, nationally representative studies reveal high prevalence of bipolar disorders emerging in early adulthood (5.5%–6.2% among 18–24-year-olds) which resolves in many by the third decade of life (3.1%–3.4% among 25–29-year-olds) [61]. This suggests that a developmentally limited form of bipolar disorder exists and may resolve spontaneously, distinguishing it from a chronic, life-long version of the disorder [61]. In addition, a cross-sectional study of 10,123 adolescents (13–18) were examined for bipolar and MDD diagnoses. 2.5% met criteria for lifetime BP I or II diagnosis, 1.7% met criteria for mania only, and rates for mania with and without depression were 2.2% and 1.3% respectively [62]. A nearly 2-fold increase in rates of mania from ages 13–14 and 17–18 was found and highlights adolescence as a peak age of onset for mania/bipolar disorder. Data indicating that mania presented separately from depression suggest that the current understanding from which diagnostic criteria are drawn requires etiologic dissection and rethinking.

## 16. How the Research Domain Criteria (RDOC) Explores Bipolar

In an attempt to clarify the discrepancy of discrete, psychiatric diagnoses which may contain some or many similar symptoms, the NIMH Research Domain Criteria (RDoC) framework seeks to instead start with discrete symptoms or “domains” and examine these domains across current psychiatric diagnoses. Using this RDoC approach, Nusslock et al., 2015 reviewed the evidence concerning unipolar and bipolar disorder within the Positive Valence Systems domain and the Approach Motivation construct, defining it as the mechanisms and processes that regulate the direction and maintenance of approach-related behaviors, which may be associated with either positive or negative emotions, directed toward innate or acquired, internal or extrinsic cues. Asymmetrical alpha frequency across the frontal cortex as measured by EEG was explored as a physiological measure of approach motivation, with an increased relative signal indicating a propensity to approach and a reduced relative signal indicating reduced approach-related motivation, and correlated with mood and anxiety symptoms [63]. Extensive support indicated that the activity of this physiological marker could denote the differential brain activation separating subjects with unipolar depression from those with bipolar depression, as well as those at risk for mania/hypomania [63]. Using the RDoC framework to characterize discrete symptoms separate from diagnoses, these same physiological markers could distinguish mania present in bipolar disorder from the anhedonia component present in unipolar depression and were hypothesized to be helpful guides in characterizing anxiety and anger as additional symptoms or symptom clusters [63].

The RDoC approach has generated considerable controversy. Proponents of the approach articulate that it was never intended to supplant diagnostic manuals such as DSM and ICD [64], but rather serve as a research approach that is agnostic to clinical diagnosis. Neuroscience-based treatment development for psychiatric disorders has stagnated over the last four decades, with molecular and neuroscientific research findings often not mapping onto clinical phenomenological approaches. Recent work suggests this may be a particularly relevant approach for biomarker studies of bipolar disorder [21]. In the context of considering mood and bipolar spectrum constructs that are not congruent with traditional categorical, descriptive diagnoses used in clinical practice, these RDoC-influenced research approaches may have particular relevance. However, at this point RDoC is not totally conducive to a clinical approach and thus neuroscience-based diagnostic categories will take many years to develop [65].

## 17. Conclusions

Though the extremes of mood known as mania and melancholia have been recognized since ancient time, the categorical diagnosis of bipolar disorder is a more modern concept. From the 18th century, recognition of a cycling disorder, including both ends of the mood spectrum to Kraepelin’s characterization of a broad mood spectrum to the modern diagnosis of episodic experiences of manic mood and depressed mood, bipolar disorder has been examined and described based on the most available clinical evidence. Arguments pertaining to the validity of the current diagnostic framework include the push for a more spectrum-based approach in which more attention is paid to sub-syndromal or sub-threshold experiences of perturbed mood. It is hoped that exploration of the varied mood states experienced by those patients with bipolar disorder can lead us toward a categorization which provides the most clinically relevant evidence to guide effective treatments. 

## Abbreviations

The following abbreviations are used in this manuscript:

BP: Bipolar
B-SNIP: Bipolar-Schizophrenia Network on Intermediate Phenotypes
DMDD: disruptive mood dysregulation disorder
DSM: Diagnostic and Statistical Manual of Mental Disorders
EEG: electroencephalograph
MDD: Major Depressive Disorder
MDE: Major Depressive Episode
MOODS-SR: Mood Spectrum Structured Interviews Self-Report
NIMH: National Institutes of Mental Health
RDoC: Research Domain Criteria
SCID: Structure Clinical Diagnostic Interview
SCI-MOODS: Mood Spectrum Structured Interviews

## References

[1] Izard C.E. (1992). Basic emotions, relations among emotions, and emotion-cognition relations. Psychol. Rev..

[2] Rosenberg E.L., Ekman P. (1994). Coherence between expressive and experiential system of emotions. Cogn. Emot..

[3] Angst J., Marneros A. (2001). Bipolarity from ancient to modern times: Conception, birth and rebirth. J. Affect. Disord..

[4] Laios K., Tsoukalas G., Kontaxaki M.I., Karamanou M., Androutsos G. (2014). Suicide in ancient greece. Psychiatriki.

[5] Angst J. (2002). Historical aspects of the dichotomy between manic—Depressive disorders and schizophrenia. Schizophrenia Res..

[6] Jablensky A. (1999). The conflict of the nosologists: Views on schizophrenia and manic-depressive illness in the early part of the 20th century. Schizophrenia Res..

[7] Zivanovic O., Nedic A. (2012). Kraepelin‘s concept of manic-depressive insanity: One hundred years later. J. Affect. Disord..

[8] American Psychiatric Association (1952). Diagnostic and Statistical Manual of Mental Disorders.

[9] American Psychiatric Association (1968). Diagnostic and Statistical Manual of Mental Disorders.

[10] Ghaemi S.N. (2013). Bipolar spectrum: A review of the concept and a vision for the future. Psychiatry Investig..

[11] American Psychiatric Association (1980). Diagnostic and Statistical Manual of Mental Disorders.

[12] American Psychiatric Association (1987). Diagnostic and Statistical Manual of Mental Disorders.

[13] American Psychiatric Association (1994). Diagnostic and Statistical Manual of Mental Disorders: Dsm-Iv.

[14] American Psychiatric Association (2000). Diagnostic and Statistical Manual of Mental Disorders.

[15] American Psychiatric Association (2013). Diagnostic and Statistical Manual of Mental Disorders.

[16] McIntyre R.S., Soczynska J.K., Cha D.S., Woldeyohannes H.O., Dale R.S., Alsuwaidan M.T., Gallaugher L.A., Mansur R.B., Muzina D.J., Carvalho A. (2015). The prevalence and illness characteristics of dsm-5-defined “mixed feature specifier“ in adults with major depressive disorder and bipolar disorder: Results from the international mood disorders collaborative project. J. Affect. Disord..

[17] Kotov R., Leong S.H., Mojtabai R., Erlanger A.C., Fochtmann L.J., Constantino E., Carlson G.A., Bromet E.J. (2013). Boundaries of schizoaffective disorder: Revisiting kraepelin. JAMA Psychiatry.

[18] Hickie I.B. (2014). Evidence for separate inheritance of mania and depression challenges current concepts of bipolar mood disorder. Mol. Psychiatry.

[19] Meyer F., Meyer T.D. (2009). The misdiagnosis of bipolar disorder as a psychotic disorder: Some of its causes and their influence on therapy. J. Affect. Disord..

[20] Roy M.A., Lanctot G., Merette C., Cliche D., Fournier J.P., Boutin P., Rodrigue C., Charron L., Turgeon M., Hamel M. (1997). Clinical and methodological factors related to reliability of the best-estimate diagnostic procedure. Am. J. Psychiatry.

[21] Clementz B.A., Sweeney J.A., Hamm J.P., Ivleva E.I., Ethridge L.E., Pearlson G.D., Keshavan M.S., Tamminga C.A. (2015). Identification of distinct psychosis biotypes using brain-based biomarkers. Am. J. Psychiatry.

[22] Hoertel N., Blanco C., Peyre H., Wall M.M., McMahon K., Gorwood P., Lemogne C., Limosin F. (2016). Differences in symptom expression between unipolar and bipolar spectrum depression: Results from a nationally representative sample using item response theory (irt). J. Affect. Disord..

[23] Benazzi F. (2006). Various forms of depression. Dialogues Clin. Neurosci..

[24] Akiskal H.S., Pinto O. (1999). The evolving bipolar spectrum: Prototypes i, ii, iii, and iv. Psychiatr. Clin. N. Am..

[25] Akiskal H.S., Bourgeois M.L., Angst J., Post R., Möller H.J., Hirschfeld R. (2000). Re-evaluating the prevalence of and diagnostic composition within the broad clinical spectrum of bipolar disorders. J. Affect. Disord..

[26] Miller S., Dennehy E.B., Suppes T. (2016). The prevalence and diagnostic validity of short-duration hypomanic episodes and major depressive episodes. Curr. Psychiatry Rep..

[27] Azorin J.M., Kaladjian A., Besnier N., Adida M., Hantouche E.G., Lancrenon S., Akiskal H. (2011). “Folie circulaire“ vs “folie a double forme“: Contribution from a french national study. Eur. Psychiatry.

[28] Koukopoulos A., Albert M.J., Sani G., Koukopoulos A.E., Girardi P. (2005). Mixed depressive states: Nosologic and therapeutic issues. Int. Rev. Psychiatry.

[29] Faedda G.L., Marangoni C., Reginaldi D. (2015). Depressive mixed states: A reappraisal of koukopoulos‘ criteria. J. Affect. Disord.

[30] Akiskal H.S., Benazzi F. (2005). Atypical depression: A variant of bipolar ii or a bridge between unipolar and bipolar ii?. J. Affect. Disord..

[31] Pacchiarotti I., Bond D.J., Baldessarini R.J., Nolen W.A., Grunze H., Licht R.W., Post R.M., Berk M., Goodwin G.M., Sachs G.S. (2013). The international society for bipolar disorders (isbd) task force report on antidepressant use in bipolar disorders. Am. J. Psychiatry.

[32] Sachs G.S., Nierenberg A.A., Calabrese J.R., Marangell L.B., Wisniewski S.R., Gyulai L., Friedman E.S., Bowden C.L., Fossey M.D., Ostacher M.J. (2007). Effectiveness of adjunctive antidepressant treatment for bipolar depression. New Engl. J. Med..

[33] Nierenberg A.A., Ostacher M.J., Calabrese J.R., Ketter T.A., Marangell L.B., Miklowitz D.J., Miyahara S., Bauer M.S., Thase M.‘kE., Wisniewski S.R. (2006). Treatment-resistant bipolar depression: A step-bd equipoise randomized effectiveness trial of antidepressant augmentation with lamotrigine, inositol, or risperidone. Am. J. Psychiatry.

[34] Tada M., Uchida H., Mizushima J., Suzuki T., Mimura M., Nio S. (2015). Antidepressant dose and treatment response in bipolar depression: Reanalysis of the systematic treatment enhancement program for bipolar disorder (step-bd) data. J. Psychiatr. Res..

[35] Benvenuti A., Miniati M., Callari A., Giorgi Mariani M., Mauri M., Dell‘Osso L. (2015). Mood spectrum model: Evidence reconsidered in the light of dsm-5. World J. Psychiatry.

[36] Merikangas K.R., Cui L., Heaton L., Nakamura E., Roca C., Ding J., Qin H., Guo W., Yao-Shugart Y., Zarate C. (2014). Independence of familial transmission of mania and depression: Results of the nimh family study of affective spectrum disorders. Mol. Psychiatry.

[37] Carlson G.A., Glovinsky I. (2009). The concept of bipolar disorder in children: A history of the bipolar controversy. Child Adolesc. Psychiatr. Clin. N. Am..

[38] Barton-Hall M. (1952). Our present knowledge about manic-depressive states. Nerv. Child.

[39] Harms E. (1952). Differential patterns of manic-depressive disease in childhood. Nerv. Child.

[40] Greding J.E., Crichton A. (1798). Medical aphorisms on melancholy, etc. An Inquiry into Mental Derangement II.

[41] Beach F. (1898). Insanity of children. J. Ment. Sci..

[42] Kraepelin E. (1921). Manic-Depressive Insanity and Paranoia.

[43] Renk K., White R., Lauer B.A., McSwiggan M., Puff J., Lowell A. (2014). Bipolar disorder in children. Psychiatry J..

[44] Baethge C., Glovinsky I., Baldessarini R.J. (2004). Manic-depressive illness in children: An early twentieth-century view by theodor ziehen (1862–1950). Introduction. Hist. Psychiatry.

[45] Kasanin J. (1931). The affective psychoses in children. Am. J. Psychiatry.

[46] Strecker E.A. (1921). The prognosis in manic depressive psychosis. N.Y. J. Med..

[47] Barrett A.M. (1952). Manic depressive psychosis in childhood. Nerv. Child.

[48] Strohl M.P. (2011). Bradley’s benzedrine studies on children with behavioral disorders. Yale J. Biol. Med..

[49] Abraham K. (1911). Notes on the psychoanalytic investigations of manic-depressive insanity and allied conditions. Selected Papers on Psychoanalysis.

[50] Klein M. (1952). Mourning and Its Elation to Manic-Depressive States.

[51] Weinberg W.A., Brumback R.A. (1976). Mania in childhood: Case studies and literature review. Am. J. Dis. Child..

[52] Goldstein B.I. (2012). Recent progress in understanding pediatric bipolar disorder. Arch. Pediatr. Adolesc. Med..

[53] Roy A.K., Lopes V., Klein R.G. (2014). Disruptive mood dysregulation disorder: A new diagnostic approach to chronic irritability in youth. Am. J. Psychiatry.

[54] Leibenluft E., Charney D.S., Towbin K.E., Bhangoo R.K., Pine D.S. (2003). Defining clinical phenotypes of juvenile mania. Am. J. Psychiatry.

[55] Brotman M.A., Kassem L., Reising M.M., Guyer A.E., Dickstein D.P., Rich B.A., Towbin K.E., Pine D.S., McMahon F.J., Leibenluft E. (2007). Parental diagnoses in youth with narrow phenotype bipolar disorder or severe mood dysregulation. Am. J. Psychiatry.

[56] Brotman M.A., Schmajuk M., Rich B.A., Dickstein D.P., Guyer A.E., Costello E.J., Egger H.L., Angold A., Pine D.S., Leibenluft E. (2006). Prevalence, clinical correlates, and longitudinal course of severe mood dysregulation in children. Biol. Psychiatry.

[57] Wiggins J.L., Brotman M.A., Adleman N.E., Kim P., Oakes A.H., Reynolds R.C., Chen G., Pine D.S., Leibenluft E. (2016). Neural correlates of irritability in disruptive mood dysregulation and bipolar disorders. Am. J. Psychiatry.

[58] Regier D.A., Narrow W.E., Clarke D.E., Kraemer H.C., Kuramoto S.J., Kuhl E.A., Kupfer D.J. (2013). Dsm-5 field trials in the united states and canada, part ii: Test-retest reliability of selected categorical diagnoses. Am. J. Psychiatry.

[59] Dickstein D.P., Towbin K.E., Van Der Veen J.W., Rich B.A., Brotman M.A., Knopf L., Onelio L., Pine D.S., Leibenluft E. (2009). Randomized double-blind placebo-controlled trial of lithium in youths with severe mood dysregulation. J. Child Adolesc. Psychopharmacol..

[60] Waxmonsky J.G., Waschbusch D.A., Belin P., Li T., Babocsai L., Humphery H., Pariseau M.E., Babinski D.E., Hoffman M.T., Haak J.L. (2016). A randomized clinical trial of an integrative group therapy for children with severe mood dysregulation. J. Am. Acad. Child Adolesc. Psychiatry.

[61] Cicero D.C., Epler A.J., Sher K.J. (2009). Are there developmentally limited forms of bipolar disorder?. J. Abnorm. Psychol..

[62] Merikangas K.R., Cui L., Kattan G., Carlson G.A., Youngstrom E.A., Angst J. (2012). Mania with and without depression in a community sample of us adolescents. Arch. Gen. Psychiatry.

[63] Nusslock R., Walden K., Harmon-Jones E. (2015). Asymmetrical frontal cortical activity associated with differential risk for mood and anxiety disorder symptoms: An rdoc perspective. Int. J. Psychophysiol..

[64] Carpenter W.T. (2016). The rdoc controversy: Alternate paradigm or dominant paradigm?. Am. J. Psychiatry.

[65] Garvey M., Avenevoli S., Anderson K. (2016). The national institute of mental health research domain criteria and clinical research in child and adolescent psychiatry. J. Am. Acad. Child Adolesc. Psychiatry.

